# Late Recurrence of Graves’ Hyperthyroidism With Thyroid Eye Disease Approximately Five Decades After Radioiodine Ablation: A Rare Clinical Scenario

**DOI:** 10.7759/cureus.103975

**Published:** 2026-02-20

**Authors:** Saraswathi Saiprasad, Narayana Swamy, Sriharika Gottipolu, Theresa Cao

**Affiliations:** 1 Endocrinology, Diabetes, and Metabolism, Baylor Scott & White Health, Fort Worth, USA; 2 Rheumatology, Baylor Scott & White Health, Fort Worth, USA; 3 Internal Medicine, Baylor Scott & White Health, Fort Worth, USA

**Keywords:** autoimmune hyperthyroidism, graves’ disease, radioiodine ablation, recurrent hyperthyroidism, thyroid ophthalmopathy recurrence

## Abstract

Radioiodine ablation (RAI) is widely regarded as definitive therapy for Graves’ disease and typically results in permanent hypothyroidism requiring lifelong thyroid hormone replacement. True recurrence of Graves’ hyperthyroidism after a prolonged post-ablative hypothyroid phase is rare and may be misinterpreted as iatrogenic thyrotoxicosis from exogenous hormone excess. We report the case of a woman in her late 60s with Graves’ disease treated with RAI in early adulthood, followed by several decades of stable hypothyroidism managed with levothyroxine, who later developed recurrent endogenous hyperthyroidism complicated by thyroid eye disease and osteoporosis. Serial thyroid function testing demonstrated persistent thyrotoxicosis despite progressive levothyroxine dose reduction and eventual discontinuation. In patients receiving thyroid hormone replacement therapy, biochemical hyperthyroidism is most commonly iatrogenic and typically resolves with dose reduction or hormone withdrawal; however, in this case, biochemical hyperthyroidism failed to recover as expected following complete discontinuation of levothyroxine, prompting further evaluation for endogenous causes of hyperthyroidism. Subsequent autoimmune testing revealed markedly elevated thyrotropin receptor antibodies and thyroid-stimulating immunoglobulins, confirming recurrent Graves’ disease. This case highlights the importance of considering endogenous hyperthyroidism in post-ablative hypothyroid patients receiving thyroid hormone replacement therapy who exhibit persistent biochemical thyrotoxicosis despite dose reduction and discontinuation, and underscores the diagnostic value of thyroid autoantibody testing in conjunction with clinical presentation in establishing disease recurrence.

## Introduction

Graves’ disease is the most common cause of persistent hyperthyroidism in adults and is managed with antithyroid medications, radioiodine ablation (RAI), or thyroidectomy [1,2]. RAI is widely regarded as a definitive treatment modality and commonly results in permanent hypothyroidism, often within the first year, necessitating lifelong levothyroxine replacement therapy. Consequently, long-term hypothyroidism following RAI is generally considered a stable and durable therapeutic outcome.

When hyperthyroidism occurs after RAI, it most commonly presents early after treatment and is typically attributed to incomplete ablation of thyroid tissue. Early persistence or recurrence has been reported in a subset of patients and is influenced by factors such as administered radioiodine dose, thyroid gland size, and baseline disease severity [3]. These presentations are generally considered manifestations of treatment failure rather than true disease recurrence.

By contrast, true late recurrence of Graves’ disease after a prolonged post-ablative hypothyroid phase is exceedingly rare. The available literature is limited primarily to isolated case reports and small case series describing recurrence occurring years to decades after successful RAI and long-term biochemical stability [4-6]. Because of its rarity, this presentation is not routinely anticipated in clinical practice.

Late recurrence presents a diagnostic challenge, particularly in patients receiving thyroid hormone replacement therapy. Suppressed thyroid-stimulating hormone (TSH) levels in this setting are most often attributed to iatrogenic hyperthyroidism from levothyroxine over-replacement. As a result, endogenous hyperthyroidism may be overlooked, especially in patients with a remote history of Graves’ disease and many years of stable post-ablative hypothyroidism. Failure to recognize this entity may delay appropriate evaluation and management, with potential clinical consequences.

In this report, we describe a woman in her late 60s who developed recurrent Graves’ hyperthyroidism several decades after RAI and long-standing post-ablative hypothyroidism managed with levothyroxine. The diagnosis was prompted by persistent biochemical thyrotoxicosis despite appropriate dose reduction and complete discontinuation of thyroid hormone replacement, in conjunction with clinical manifestations of hyperthyroidism, including tremors and persistent sinus tachycardia, as well as the development of thyroid eye disease and progressive bone loss. This case highlights the importance of diagnostic vigilance in post-ablative hypothyroid patients with discordant biochemical and clinical findings and underscores the diagnostic value of thyroid autoantibody testing in conjunction with clinical presentation in establishing disease recurrence.

## Case presentation

A woman in her late 60s presented to the endocrinology clinic for evaluation of tremors and biochemical hyperthyroidism. Her body mass index was 24.22 kg/m² (weight 56 kg). She had a remote history of Graves’ disease diagnosed in her 20s, for which she underwent RAI with 5 mCi of I-131. She subsequently developed hypothyroidism in her 40s and remained on stable levothyroxine therapy (88 µg daily) for several decades with documented biochemical euthyroidism. She was a former smoker who quit approximately 40 years prior to presentation.

Approximately six months prior to presentation, she developed progressive symptoms consistent with thyrotoxicosis, including tremors, heat intolerance, restlessness, and persistent sinus tachycardia, with resting heart rates near 100 beats per minute. Initial laboratory testing demonstrated a suppressed TSH level of <0.005 µIU/mL (reference range: 0.590-4.780 µIU/mL) with an elevated free thyroxine (FT4) of 2.54 ng/dL (reference range: 0.92-1.68 ng/dL) while taking 88 mcg of levothyroxine daily. This represented a marked deviation from prior stable thyroid function, including a TSH of 1.10 µIU/mL (reference range: 0.590-4.780 µIU/mL) two years earlier and 0.94 µIU/mL (reference range: 0.590-4.780 µIU/mL) one year earlier on the same levothyroxine dose, both consistent with adequate replacement and biochemical euthyroidism (Table 1).

**Table 1:** Thyroid function trends during levothyroxine dose reduction and diagnostic reassessment Reference ranges: TSH: 0.590-4.780 µIU/mL; free T4: 0.92-1.68 ng/dL FT4, free thyroxine; TFT, thyroid function test; TRAb, thyrotropin receptor antibody; TSH, thyroid-stimulating hormone; TSI, thyroid-stimulating immunoglobulin

Clinical time point	Levothyroxine dose	TSH (µIU/mL)	Free T4 (ng/dL)	Key clinical notes
Two years prior to abnormal labs	88 mcg daily	1.10	Not available	Euthyroid on levothyroxine
One year prior to abnormal labs	88 mcg daily	0.94	Not available	Adequate replacement; euthyroid
Initial abnormal labs (six months prior to presentation)	88 mcg daily	<0.005	2.54	Biochemical hyperthyroidism detected
Dose reduction #1	Reduced from 88 to 75 mcg	<0.005	2.54	No biochemical improvement
Dose reduction #2	Reduced from 75 to 50 mcg	<0.005	2.09	Persistent hyperthyroidism
Endocrinology clinic visit	Levothyroxine discontinued	<0.005	2.07	TFTs not recovering as expected; endogenous hyperthyroidism suspected
Autoimmune evaluation	Not applicable	Not applicable	Not applicable	Markedly elevated thyroid antibodies: TRAb 32.88 IU/L (≤2.00 IU/L); TSI 210% (<140%)

Given concern for iatrogenic hyperthyroidism, her primary care physician initiated stepwise reductions in levothyroxine dosing. However, repeat testing continued to demonstrate persistent biochemical hyperthyroidism, with TSH remaining <0.005 µIU/mL (reference range: 0.590-4.780 µIU/mL) and FT4 of 2.54 ng/dL (reference range: 0.92-1.68 ng/dL) following dose reduction to 75 mcg daily, and TSH remaining <0.005 µIU/mL (reference range: 0.590-4.780 µIU/mL) with FT4 of 2.09 ng/dL (reference range: 0.92-1.68 ng/dL) after further reduction to 50 mcg daily (Table 1). Due to persistent biochemical hyperthyroidism despite dose reduction, she was referred to endocrinology for further evaluation. At the time of her endocrinology visit, repeat testing again demonstrated suppressed TSH (<0.005 µIU/mL; reference range: 0.590-4.780 µIU/mL) with an FT4 of 2.07 ng/dL (reference range: 0.92-1.68 ng/dL), and levothyroxine was discontinued, as these findings were not consistent with iatrogenic hyperthyroidism and raised concern for endogenous hyperthyroidism. She denied biotin use or intake of supplements known to interfere with thyroid function testing. Subsequent autoimmune evaluation revealed markedly elevated thyroid antibodies, including thyrotropin receptor antibodies (TRAbs) of 32.88 IU/L (≤2.00 IU/L) and thyroid-stimulating immunoglobulins (TSIs) of 210% (<140%), confirming recurrent Graves’ disease. Thyroid function trends during levothyroxine dose reduction and diagnostic reassessment are summarized in Table 1.

Post-levothyroxine discontinuation and antithyroid therapy

Serial thyroid function testing following levothyroxine discontinuation demonstrated persistent TSH suppression with minimal early biochemical recovery at three and six weeks. FT4 measurements were not available at these early post-discontinuation time points. At eight weeks, FT4 improved to 1.38 ng/dL (reference range: 0.92-1.68 ng/dL); however, TSH remained suppressed. In the setting of ongoing hyperthyroid symptoms despite the absence of thyroid hormone therapy and markedly elevated TRAbs and TSIs, antithyroid therapy was initiated. Methimazole was started at a low dose of 2.5 mg daily, with close clinical and biochemical monitoring. Thyroid function trends after levothyroxine discontinuation and during methimazole therapy are summarized in Table 2.

**Table 2:** Thyroid function trends after levothyroxine discontinuation and methimazole therapy Reference ranges: TSH: 0.590-4.780 µIU/mL; free T4: 0.92-1.68 ng/dL FT4, free thyroxine; TRAb, thyrotropin receptor antibody; TSH, thyroid-stimulating hormone; TSI, thyroid-stimulating immunoglobulin

Time point	Therapy	TSH (µIU/mL)	Free T4 (ng/dL)	Key clinical notes
Three weeks post-levothyroxine discontinuation	None	<0.005	Not available	Persistent TSH suppression following hormone withdrawal
Six weeks post-levothyroxine discontinuation	None	<0.01	Not available	Minimal biochemical recovery
Eight weeks post-levothyroxine discontinuation	None → methimazole initiated	<0.05	1.38	Total T3: 189 ng/dL (80-200) - high normal. Persistent hyperthyroid symptoms with markedly elevated thyroid antibodies (TRAb 32.88 IU/L; TSI 210%); methimazole initiated at 2.5 mg daily
Six weeks after methimazole initiation	Methimazole 2.5 mg daily	<0.005	1.27	Persistent TSH suppression; methimazole dose increased
Six weeks after the preceding dose increase	Methimazole 5 mg daily	0.03	0.88	Biochemical improvement; dose continued
12 weeks after the preceding dose increase	Methimazole 5 mg daily	0.02	1.13	Delayed TSH recovery with a relative rise in free T4; methimazole dose increased by an additional 5 mg per week
Most recent laboratory evaluation, obtained six weeks after the preceding dose increase	Methimazole 5 mg daily for 6 days/week and 10 mg on 1 day/week	0.88	0.81	Normalization of TSH with free T4 slightly below the reference range, prompting a reduction of methimazole back to 5 mg daily to avoid overtreatment

Six weeks after initiation of methimazole, FT4 remained within the reference range at 1.27 ng/dL (reference range: 0.92-1.68 ng/dL); however, TSH demonstrated further suppression (<0.005 µIU/mL; reference range: 0.590-4.780 µIU/mL), prompting an increase in the methimazole dose to 5 mg daily. Repeat testing six weeks later showed biochemical improvement, with TSH rising to 0.03 µIU/mL (reference range: 0.590-4.780 µIU/mL) and FT4 decreasing to 0.88 ng/dL (reference range: 0.92-1.68 ng/dL), and the methimazole dose was continued. On subsequent testing six weeks thereafter, TSH remained low at 0.02 µIU/mL (reference range: 0.590-4.780 µIU/mL) with an FT4 of 1.13 ng/dL (reference range 0.92-1.68 ng/dL). Given the limited recovery of TSH and a rising FT4 level, the methimazole dose was modestly increased by an additional 5 mg per week, using a regimen of 5 mg daily for six days of the week and 10 mg on one day per week, with plans to repeat thyroid function testing in six weeks.

On the most recent laboratory evaluation, obtained six weeks after the preceding dose increase, thyroid function tests demonstrated a TSH of 0.88 µIU/mL (reference range: 0.590-4.780 µIU/mL) with a free T4 of 0.81 ng/dL (reference range: 0.92-1.68 ng/dL), reflecting recovery of TSH and a favorable biochemical response to the modest dose escalation. As the free T4 level had fallen below the lower limit of the reference range, methimazole was reduced back to 5 mg daily to avoid overtreatment. The patient reported continued improvement in hyperthyroid symptoms, consistent with biochemical stabilization. The longitudinal biochemical response following levothyroxine discontinuation and methimazole dose adjustments is detailed in Table 2.

Ophthalmic findings

During the course of evaluation, the patient developed ocular symptoms consistent with Graves’ ophthalmopathy, further supporting the diagnosis of recurrent Graves’ disease, and she was referred to neuro-ophthalmology. At the time of writing this report, her ocular symptoms were improving with ongoing methimazole therapy.

Imaging findings

**Figure 1:**
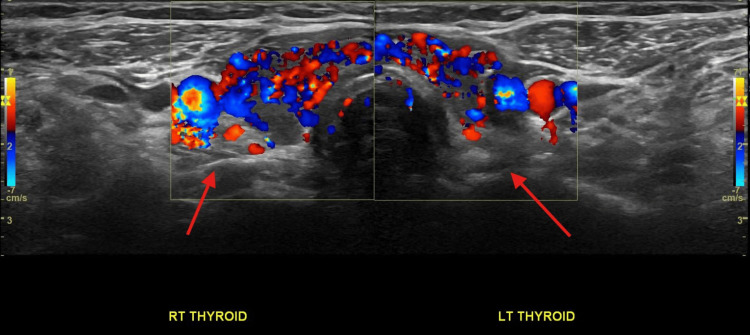
Thyroid ultrasound findings Color Doppler image demonstrating increased parenchymal vascularity, consistent with inflammatory thyroid disease.

**Figure 2:**
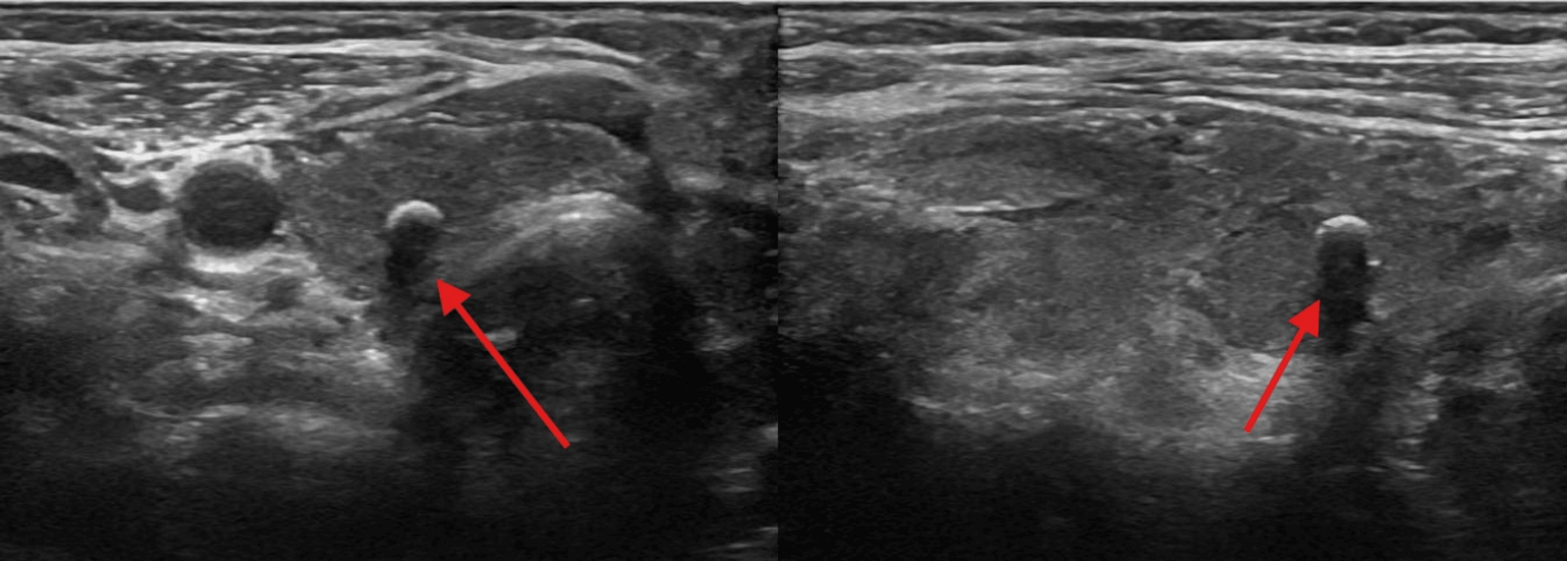
Thyroid ultrasound findings Transverse ultrasound image showing a well-circumscribed nodule in the right thyroid lobe.

**Figure 3:**
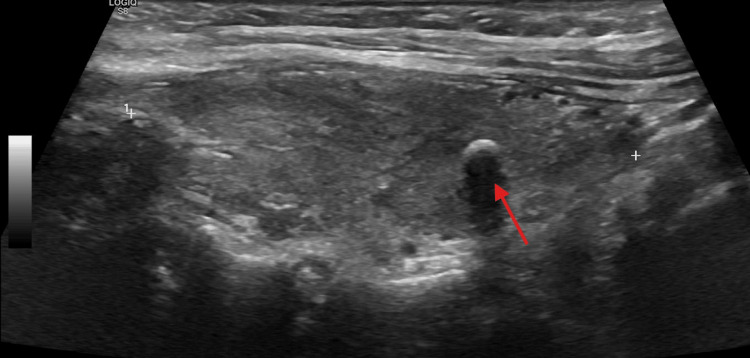
Thyroid ultrasound findings Sagittal ultrasound image showing a well-circumscribed nodule in the right thyroid lobe.

**Figure 4:**
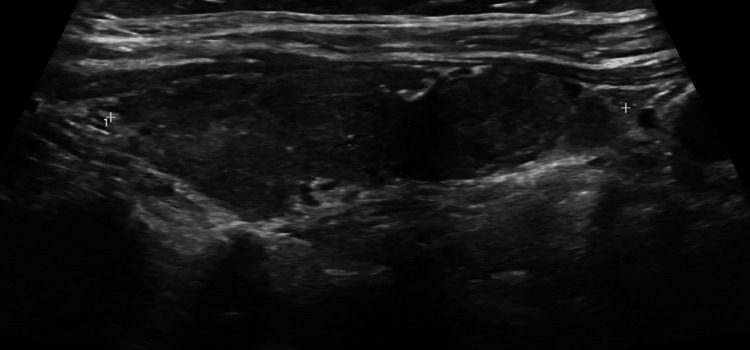
Thyroid ultrasound findings Sagittal ultrasound image of the left thyroid lobe demonstrated no dominant or discrete nodules.

Thyroid ultrasound demonstrated a post-ablative gland with markedly heterogeneous parenchyma, diffusely decreased echogenicity, and increased vascularity on Doppler imaging, findings consistent with inflammatory thyroid disease (Figure 1). Multiple bilateral subcentimeter nodules were identified. In the right thyroid lobe, a 0.8 × 0.6 × 0.7 cm TI-RADS 5 nodule and a second 0.4 × 0.4 × 0.4 cm TI-RADS 5 nodule were present (Figure 2, Figure 3). In the left thyroid lobe, two subcentimeter TI-RADS 4 nodules measuring 0.5 × 0.3 × 0.3 cm and 0.4 × 0.6 × 0.3 cm were noted. Sagittal ultrasound imaging of the left thyroid lobe demonstrated no dominant or discrete nodules (Figure 4). None of the nodules met the size criteria for biopsy, despite TI-RADS 4 and 5 features, as all were subcentimeter. Overall, the ultrasound findings were dominated by diffuse parenchymal changes consistent with thyroiditis.

Bone health

**Figure 5:**
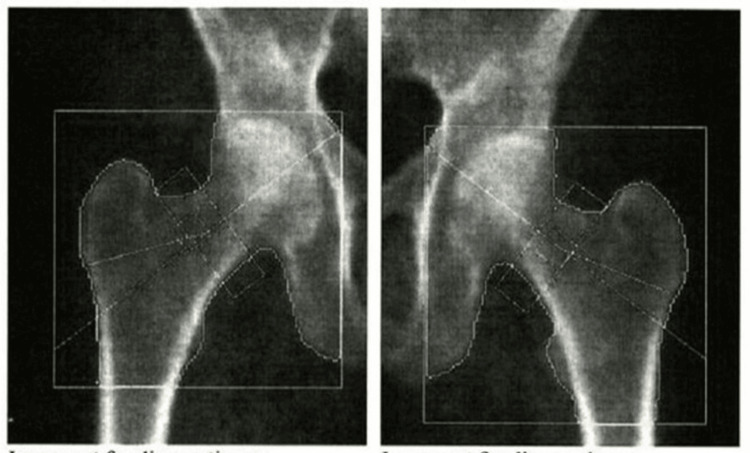
Bilateral hip DXA scans Right and left hip DXA scan images were obtained using a Hologic densitometer (Hologic Inc., Marlborough, MA, USA). The right hip is shown on the left side of the image and the left hip on the right, in standard radiologic convention. Quantitative DXA measurements are presented separately in Table 3. DXA, dual-energy X-ray absorptiometry

**Figure 6:**
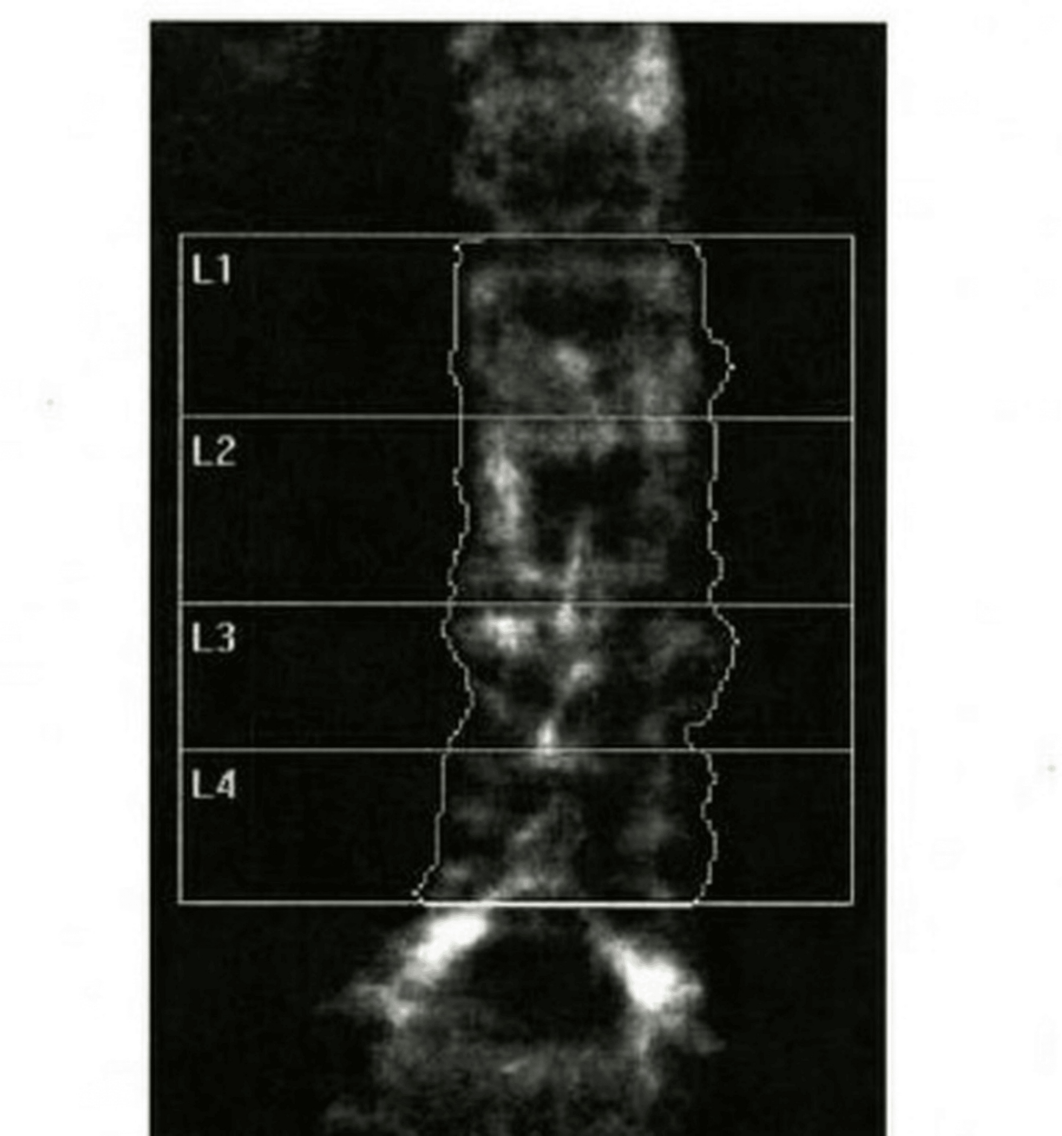
Anterior-posterior lumbar spine DXA scan (L1-L4) The lumbar spine DXA scan (L1-L4) was acquired using a Hologic densitometer (Hologic Inc.). Quantitative DXA measurements are presented separately in Table 4. DXA, dual-energy X-ray absorptiometry

Dual-energy X-ray absorptiometry (DXA) findings are contextualized relative to the timing of recurrent hyperthyroidism, with acknowledgment that bone loss in this patient is likely multifactorial, including age, postmenopausal status, and prior thyroid disease. The patient had a long-standing history of osteopenia that had progressed to osteoporosis. DXA performed approximately one year prior demonstrated osteoporosis at both hips. The right femoral neck had a T-score of -2.6 (bone mineral density (BMD): 0.556 g/cm²), and the left femoral neck had a T-score of -2.7 (BMD: 0.550 g/cm²). The right and left total femur T-scores were -2.5 (BMD: 0.635 and 0.634 g/cm², respectively) (Figure 5, Table 3). The anteroposterior lumbar spine demonstrated osteopenia with a T-score of -1.6 (BMD: 0.871 g/cm²) (Figure 6, Table 4). Given the patient’s age, postmenopausal status, hip osteoporosis, and recurrent hyperthyroidism, each independently associated with increased fracture risk, treatment with calcium, vitamin D₃, and intravenous zoledronic acid was initiated.

**Table 3:** Femoral neck and total hip DXA results summary DXA performed on a Hologic densitometer showed femoral neck BMD of 0.550-0.556 g/cm² (T-score -2.6 to -2.7; Z-score -1.0) and total hip BMD of 0.634-0.635 g/cm² (T-score -2.5; Z-score -1.1 to -1.2), consistent with osteoporosis. BMC, bone mineral content; BMD, bone mineral density; DXA, dual-energy X-ray absorptiometry

Region	Side	Area (cm²)	BMC (g)	BMD (g/cm²)	T-score	Z-score
Femoral neck	Left	4.62	2.54	0.550	-2.7	-1.0
Femoral neck	Right	4.81	2.67	0.556	-2.6	-1.0
Femoral neck	Mean	4.71	2.60	0.553	-2.7	-1.0
Total hip	Left	34.9	22.14	0.634	-2.5	-1.2
Total hip	Right	32.15	20.42	0.635	-2.5	-1.1
Total hip	Mean	33.52	21.28	0.635	-2.5	-1.1

**Table 4:** Spine DXA results summary DXA performed on a Hologic densitometer showed total lumbar spine (L1-L4) BMD of 0.871 g/cm², with a T-score of -1.6 and a Z-score of 0.3, consistent with osteopenia. BMC, bone mineral content; BMD, bone mineral density; DXA, dual-energy X-ray absorptiometry

Region	Area (cm²)	BMC (g)	BMD (g/cm²)	T-score	Z-score
L1	13.51	11.77	0.871	-1.1	0.6
L2	14.05	12.47	0.888	-1.3	0.6
L3	11.18	9.98	0.893	-1.7	0.3
L4	12.66	10.58	0.835	-2.1	0.0
Total (L1-L4)	51.4	44.79	0.871	-1.6	0.3

## Discussion

Late recurrence of Graves’ disease after RAI

Graves’ disease is the most common cause of persistent hyperthyroidism in adults, and RAI remains a widely used definitive treatment modality [1,2]. Following RAI, permanent hypothyroidism commonly develops, often within the first year, necessitating lifelong levothyroxine replacement therapy [1]. When hyperthyroidism persists or recurs after RAI, it most commonly presents early after treatment and is generally attributed to incomplete ablation of thyroid tissue. Such early persistence or recurrence has been reported in a subset of patients and is influenced by factors including administered radioiodine dose and baseline disease severity [3].

In contrast, true late recurrence of Graves’ disease after a prolonged post-ablative hypothyroid phase is exceedingly rare. The available literature consists largely of isolated case reports and small case series describing recurrence occurring years to decades after successful RAI and long-term biochemical stability [4,6]. Reported intervals between RAI and late recurrence extend from several years to more than three decades, underscoring that autoimmune reactivation may occur long after apparent disease remission [4,6].

The present case illustrates this uncommon phenomenon, with recurrent endogenous hyperthyroidism developing several decades after RAI and after a prolonged period of stable post-ablative hypothyroidism. This presentation highlights that, although rare, late recurrence remains a clinically relevant consideration even many years after definitive therapy.

Diagnostic considerations in post-ablative thyrotoxicosis

In patients with prior RAI who are receiving levothyroxine therapy, suppressed TSH levels are most frequently attributed to exogenous thyroid hormone over-replacement. Accordingly, dose reduction or discontinuation of levothyroxine is typically the initial management step. However, persistent biochemical hyperthyroidism despite serial dose reductions and complete withdrawal of thyroid hormone replacement should prompt evaluation for endogenous causes of thyrotoxicosis, including recurrent Graves’ disease, toxic multinodular goiter, toxic adenoma, and thyroiditis [1].

In this case, failure of thyroid function tests to normalize following complete levothyroxine discontinuation was a critical diagnostic clue. Measurement of TRAbs and TSIs played a decisive role in confirming disease recurrence. The markedly elevated TRAb (32.88 IU/L) and TSI (210%) levels in this patient confirmed active autoimmune stimulation of residual thyroid tissue and effectively excluded alternative etiologies such as thyroiditis.

Pathophysiology of late recurrence after RAI

The mechanisms underlying late recurrence of Graves’ disease following RAI are incompletely understood but are likely multifactorial. RAI induces thyroid follicular cell destruction through beta radiation; however, complete eradication of thyroid tissue is uncommon, particularly in patients treated with lower radioiodine doses that were frequently used in earlier decades [3]. Early persistence of hyperthyroidism following RAI is typically attributed to incomplete ablation of thyroid tissue and has been reported in a subset of patients, with risk influenced by administered radioiodine dose, thyroid gland size, and baseline disease severity [3]. As a result, small amounts of residual thyroid tissue may persist and remain biologically responsive to immune stimulation.

Graves’ disease is mediated by stimulatory TRAbs that activate the TSH receptor and drive unregulated thyroid hormone synthesis. Although TRAb titers typically decline following RAI and may become undetectable within one to three years, longitudinal studies demonstrate that antibody levels can fluctuate over time. Reemergence or amplification of TRAb production years or decades later suggests that RAI does not eliminate the underlying autoimmune predisposition. Once TRAb levels exceed a functional threshold, even minimal residual thyroid tissue may produce clinically significant hyperthyroidism.

Thyroid eye disease and systemic implications

The development of thyroid eye disease in this patient further supports autoimmune disease reactivation. Graves’ ophthalmopathy occurs in a substantial proportion of patients with Graves’ disease, with moderate-to-severe manifestations reported in a minority [7]. While ophthalmopathy most often presents concurrently with initial hyperthyroidism, it may also develop or worsen during disease recurrence, even many years after initial treatment.

The pathogenesis of thyroid eye disease involves shared antigen expression between thyroid follicular cells and orbital fibroblasts, particularly the thyrotropin receptor and insulin-like growth factor-1 receptor. Reactivation of TRAb production can therefore simultaneously stimulate residual thyroid tissue and orbital fibroblasts, explaining the concurrent recurrence of hyperthyroidism and eye disease observed in this case [7].

Hyperthyroidism is also associated with important systemic complications. Cardiovascular morbidity, particularly atrial fibrillation, is well described in older patients with untreated thyrotoxicosis. In addition, prolonged hyperthyroidism accelerates bone turnover and increases fracture risk, particularly in postmenopausal women [8]. These risks were especially relevant in this patient, given her underlying osteoporosis, underscoring the importance of timely recognition and treatment of recurrent disease.

Strengths and limitations

A key strength of this case is the detailed longitudinal documentation of thyroid function spanning several decades, which allowed clear differentiation between long-standing post-ablative hypothyroidism and true late recurrence of Graves’ disease. The stepwise approach to levothyroxine dose reduction, complete withdrawal, and subsequent reassessment provided a robust framework to exclude iatrogenic hyperthyroidism before pursuing evaluation for endogenous causes. In addition, confirmation of disease recurrence with markedly elevated TRAbs and TSIs, along with concordant clinical findings including thyroid eye disease, strengthens the diagnostic certainty.

This report also has limitations inherent to single-patient case studies. Thyroid autoantibody measurements from the patient’s initial Graves’ disease diagnosis several decades earlier were not available, reflecting historical practice patterns and limiting direct longitudinal comparison of antibody titers. Direct assessment of residual functioning thyroid tissue with repeat radioiodine uptake scanning was not performed, and the exact immunologic trigger for late disease reactivation cannot be determined. Furthermore, the rarity of this presentation limits generalizability and precludes estimation of true recurrence risk following RAI.

Main clinical lesson

The principal clinical lesson from this case is that persistent biochemical thyrotoxicosis in post-ablative hypothyroid patients should not be automatically attributed to exogenous thyroid hormone excess. Failure of thyroid function tests to normalize following appropriate dose reduction and discontinuation of levothyroxine should prompt evaluation for endogenous hyperthyroidism, including measurement of thyroid autoantibodies. Awareness of this rare but clinically important phenomenon may facilitate earlier recognition, appropriate treatment, and prevention of systemic complications associated with unrecognized recurrent Graves’ disease.

## Conclusions

This case demonstrates that RAI does not invariably confer lifelong protection against recurrence of Graves’ disease. In this patient, disease recurrence occurred nearly five decades after initial treatment, following a prolonged period of stable post-ablative hypothyroidism requiring levothyroxine replacement. Such an extended latency underscores the exceptional rarity of this phenomenon and highlights that autoimmune thyroid disease may remain immunologically active despite apparent long-term clinical remission.

From a clinical standpoint, this case reinforces the importance of maintaining a high index of suspicion for endogenous hyperthyroidism in post-ablative hypothyroid patients when thyroid function tests fail to normalize as expected with levothyroxine dose reduction or discontinuation. Persistent biochemical thyrotoxicosis in this setting should prompt further evaluation, including measurement of thyroid autoantibodies, rather than attribution solely to exogenous hormone excess. Recognition of this rare but clinically significant presentation may facilitate timely diagnosis, appropriate management, and prevention of long-term complications in similar patients.
